# Existing Filtration Treatment on Drinking Water Process and Concerns Issues

**DOI:** 10.3390/membranes13030285

**Published:** 2023-02-27

**Authors:** Mashitah Che Razali, Norhaliza Abdul Wahab, Noorhazirah Sunar, Nur Hazahsha Shamsudin

**Affiliations:** 1 Faculty of Electrical Engineering, Universiti Teknikal Malaysia Melaka, Hang Tuah Jaya, Durian Tunggal, Melaka 76100, Malaysia; 2 Faculty of Electrical Engineering, Universiti Teknologi Malaysia, Johor Bahru 81310, Malaysia

**Keywords:** filtration, drinking water, membrane fouling, fouling prevention, fouling prediction, fouling control

## Abstract

Water is one of the main sources of life’s survival. It is mandatory to have good-quality water, especially for drinking. Many types of available filtration treatment can produce high-quality drinking water. As a result, it is intriguing to determine which treatment is the best. This paper provides a review of available filtration technology specifically for drinking water treatment, including both conventional and advanced treatments, while focusing on membrane filtration treatment. This review covers the concerns that usually exist in membrane filtration treatment, namely membrane fouling. Here, the parameters that influence fouling are identified. This paper also discusses the different ways to handle fouling, either based on prevention, prediction, or control automation. According to the findings, the most common treatment for fouling was prevention. However, this treatment required the use of chemical agents, which will eventually affect human health. The prediction process was usually used to circumvent the process of fouling development. Based on our reviews up to now, there are a limited number of researchers who study membrane fouling control based on automation. Frequently, the treatment method and control strategy are determined individually.

## 1. Introduction

The quality of drinking water resources is being enthusiastically addressed around the world since it is essential to health and development issues. Due to uncontrolled industrial waste and low public awareness, water pollutants can be discharged either directly or indirectly to water resources such as lakes, ponds, rivers, seawater, and groundwater, which later become contaminated. The contaminated or poor quality of drinking water can cause various infectious diseases and negatively impact our overall health [1]. According to the World Health Organization (WHO), contaminated drinking water can cause serious diseases such as diarrhea, cholera, dysentery, hepatitis A, typhoid, and polio [2]. It is estimated that around 502,000 people die each year from diarrhea due to unsafe drinking water. The quality of water resources has been gradually depreciating due to industrialization and urbanization [3]. It has become a crucial problem due to the difficulty of meeting effluent quality standards with conventional treatment processes [4,5,6]. Good-quality drinking water helps people achieve maximum body health and well-being.

To obtain high-quality drinking water, a good and reliable water treatment process is desirable. Traditional drinking water treatment includes five common units such as coagulation, flocculation, sedimentation, filtration, and disinfection [7,8,9]. More than ten decades ago, the only treatment processes used in municipal and industrial water treatment were conventional filtration, such as clarification and granular media filtration, and chlorination methods. However, in the past twenty years, industrial water has shown high interest in the implementation of advanced water treatment technologies, particularly for water purification technologies such as membrane filtration, ultraviolet irradiation, the advanced oxidation process (AOP), ion exchange, and biological filtration for the removal of water contaminants in drinking water [10]. As of today, the latest water purification technologies are nanotechnology, acoustic nanotube, photocatalytic water purification, aquaporin inside^TM^, and automatic variable filtration. The use of technologies in water treatment is mainly due to three main reasons: a new standard for water quality, an increase in water contamination, and cost. Certainly, the new technology to be introduced should provide more advantages over the conventional treatment processes, such as lower operation and maintenance costs, being more efficient and simple to operate, having higher effluent quality and a high degree of reliability, having lower waste production, and most importantly, meeting regulatory requirements.

This paper identifies and reviews some of the available technologies ‘often’ used in drinking water treatment. Many of them are certainly not new to the water industry, but their application has been limited due to many circumstances, which are highlighted in this paper. This review focuses on membrane filtration technology and its application to municipal and industrial water systems. This review was motivated to establish an understanding of the related issues that come up in the drinking water treatment process.

We gather information about the available filtration systems, with a focus on the differences between conventional and membrane filtration, from these studies. Membrane filtration problems such as fouling phenomena, membrane cleaning, fouling prediction, and fouling prevention are discussed thoroughly. We also discussed the consequences of this review for the selection of a control strategy to overcome the problem in drinking water treatment, particularly due to the fouling phenomena. The goal is to organize and summarize most of the work and to identify the research focus and the trends in the literature on filtration treatment methods for drinking water processes.

## 2. Available Drinking Water Treatment Technologies

In general, the treatment technologies for treating water depend on the type of raw intake water that comes from various water sources, such as surface water and groundwater. The existing filtration treatments that are covered in this section are divided into conventional and advanced methods. Some of the available drinking water filtration treatment technologies, both conventional and advanced, as well as their concerns, are described.

### 2.1. Conventional Treatment

Conventional treatment is one of the popular approaches that has been used for water and wastewater treatment systems, where it involves several processes, including bar screening, grit removal, pre-oxidation, coagulation, flocculation, sedimentation, rapid/slow sand, granular active carbon filtration, and/or disinfection [11]. These processes can remove various solid sizes and organic matter from the liquid phase. It is also able to contribute to the reduction of microorganisms that cause concern for public health. There are several types of conventional filtration treatments, such as simple screen filters, slow and fast sand filters, diatom filters, and charcoal filters. The effect of filter media on the filtration process needs to be considered when designing the filtration unit. Additionally, the design of the backwash filter needs to be taken into account when high turbidity in effluent water increases head losses and requires long filtration operations [12,13].

Many studies have been performed to investigate the effectiveness of conventional filtration in treating drinking water. The previous study of the removal of diclofenac from drinking water is reported by Rigobello et al. [14], where the conventional sand filter is compared with granular activated carbon (GAC) filtration. The results showed that a sand filter could not effectively remove diclofenac, whereas a combination of a sand filter and GAC filtration could remove diclofenac with ≥99.7% efficiency. A slow sand filter and charcoal filter have been used in the study by Murugan and Ram [15]. The application of a slow sand filter can help in the reduction of water turbidity and prevent fouling at the reactor tubes. The charcoal filter is used to help in the absorption of heavy metals that are present in the water. In this work, slow sand filters require periodic removal of the microbial layer, while charcoal must be replaced in the filter every month as there are no indications that the charcoal has reached its breakthrough.

Zheng et al. [16] investigate the use of a slow sand filter as a pre-treatment for the removal of organic foulants in secondary effluent. The investigation was conducted with different filtration rates and showed that the proposed pre-treatment can effectively control the fouling rate at low filtration rates with respect to biopolymer removal and cycle time. Another study on the effect of a flow configuration based on a slow sand filter was performed by Sabogal-Paz et al. [17], where a comparison study was performed for the household system between intermittent and continuous flows. The authors observe that the flow configuration of a slow sand filter cannot be applied as a single treatment because it is not able to remove the organic foulants effectively. The work proposed by Ahammed and Darva [18] investigates the effect of a modified slow sand filter by introducing a thin layer of iron oxide-coated sand. The performance of the proposed method is measured based on its capability to remove bacteria and turbidity. Results showed that the modified slow sand filter was able to increase the removal rate of bacteria, but there was no significant reduction in turbidity. Work by Mizuta et al. [19] presents bamboo powder charcoal and activated carbon filtration in the removal of nitrate and nitrogen from drinking water. The results showed that bamboo powder charcoal filtration was able to provide higher adsorption and less influence on temperature compared to activated carbon filtration. Bamboo charcoal filtration was studied by Zhang et al. [20] to remove microcystin-LR from drinking water. In this study, bamboo charcoal filtration was modified with chitosan, and the results indicate that the applied treatment was able to effectively remove the microcystin-LR, especially when the amount of bamboo charcoal was increased.

Based on previous studies of conventional treatment methods, it is clear that the method is incapable of producing satisfactory effluent quality. Most of the treatments require either modification or combination with other methods, which is costly due to frequent maintenance. Moreover, this treatment is considered economically unbeneficial for developing countries [21], where the treatments require a long operating period and a large footprint [22]. Due to the importance of having safe and healthy water, water utilities have started to consider alternative treatment technologies to traditional drinking water treatment.

### 2.2. Advanced Treatment

Here, several advanced treatments of water technologies, particularly for water purification technologies such as membrane filtration, ultraviolet irradiation, the advanced oxidation process, ion exchange, and biological filtration, are discussed. Recently, membrane filtration is increasingly being accepted and implemented in drinking water treatment plants [23]. Membrane technology is widely used in filtration systems, particularly for the removal of particulate matter in solid-liquid separation processes [24,25]. Moreover, the combination of membrane technology with a bioreactor is called a membrane bioreactor, and this technology has proven its high capacity for the removal of pollutants in water and wastewater treatment processes [26,27]. The main issue in membrane filtration is the fouling phenomenon, which, if not prevented, will affect the overall filtration performance in the long run.

Another advanced technology that is primarily used in drinking water is ultraviolet (UV) irradiation technology [28]. UV irradiation is used as a disinfection process and is commonly designed with a series of UV lamps so that the microorganisms in the water will be inactivated when exposed to UV light [29]. Although UV irradiation is a promising disinfection technology due to its compactness and low cost, it faces a challenge due to its reliance on electrical component sensitivity [30], which can result in high failure rates.

**Figure 1:**
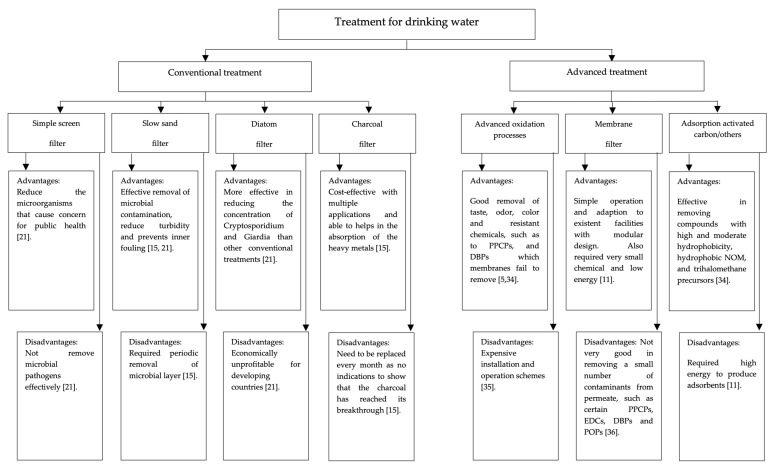
Available treatment for drinking water [5,11,15,21,34,35,36].

The advanced oxidation process (AOP) is another technology generally applied in water treatment. The AOP includes several processes that produce hydroxyl radicals for the oxidation of organic and inorganic water impurities [31]. Among the three main AOP processes are ozone, ozone with hydrogen peroxide addition, and UV irradiation with hydrogen peroxide addition. Each of the processes has its challenges and will not be discussed in detail here. To summarize, AOP can provide multiple uses in water treatment, such as color, oxidation of synthetic organic chemicals, taste and odor, and many more. However, the complexity of AOPs in terms of chemical reactions between processes makes it hard to achieve an optimum treatment system design [32]. The next advanced water treatment is ion exchange (IX) technology. This technology was previously limited to only softening water for use in water treatment plants. However, the limits are now also being set on several inorganic chemicals, making the IX a more interesting technology to explore in water treatment applications. Lastly, biological filtration is another type of advanced treatment in water technology. The filtration is based on biological processes, which are different from the previously mentioned technologies that are based on physical and/or chemical processes. Works by Wang et al. [33] claim this biological filtration is the most effective process to produce biologically stable water. However, there are still unanswered issues regarding the proper design and implementation of biological filtration, particularly in terms of the size and type of filter media to be used. Figure 1 summarizes the conventional and advanced filtration methods for drinking water treatment.

### 2.3. Hybrid Treatment

**Figure 2:**
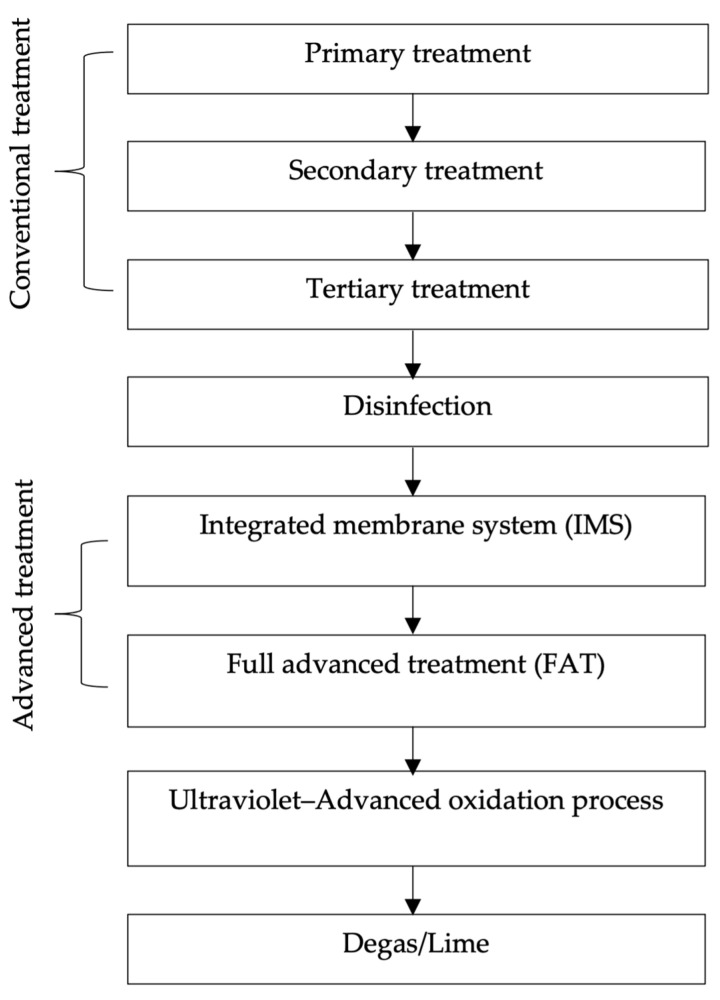
Industry-standard potable reuse plant.

In general, most industrial drinking water treatments still involve conventional and advanced treatment processes [8]. Figure 2 shows an example of industry-standard potable reuse water plants that involve conventional and advanced treatment processes [8]. In the primary treatment, the sedimentation of solid waste is performed. Water from secondary and tertiary treatment can be used for potable and non-potable reuse applications. The secondary treatment involves biological processes (e.g., the activated sludge process), and the tertiary treatment involves physical and/or chemical processes. For the disinfection process, chlorine is used to disinfect water to kill bacteria, parasites, and viruses in drinking water [37]. Alternatively, disinfectants such as chlorine dioxide, ozone, and ultraviolet radiation are also used. In advanced treatment, the integrated membrane system (IMS) and full advanced treatment (FAT) are implemented. The IMS uses a low-pressure membrane filtration process either microfiltration (MF) or ultrafiltration (UF). Meanwhile, FAT applies called either nanofiltration (NF) or reverse osmosis (RO), which are high-pressure membrane filtration processes. The application of IMS can provide high efficacy in the removal rate of particulate matter, microbial pathogens, and natural organic matter, whereas FAT is capable of removing magnificently organic–inorganic dissolved constituents such as salts and organic chemicals that are impossible to be removed by IMS. Ultraviolet and advanced oxidation processes act as post-treatment disinfection. In this stage, it will break down small neutral organic compounds that pass-through FAT. The final stage is known as degassing and lime dosing, which act as a water stabilizer and increase the pH and alkalinity of the water. The industry standard potable reuse water plant shown in Figure 2 can meet the specification for drinking water quality, but there are several drawbacks, including a large footprint, high capital cost, and high energy consumption, which make it essential to discover another technology that can overcome the drawbacks [38].

The conventional design of the drinking water treatment process includes five common units, and four of them (coagulation, flocculation, sedimentation, and filtration) are the lines that remove suspended particles from surface water treatment plants. Filtration is the final step in the removal of suspended particles, and without it, the plants are considered untreatable. Therefore, proper control, design, and implementation of the filtration operation unit are crucial to improving the effluent quality and reducing the risk of waterborne diseases. The next section then focuses on a review of numerous types of membrane filtration technologies. The advantages and disadvantages of each type of filtration are also discussed, and this will provide some hints for researchers on how to choose the most suitable membrane filtration for their applications.

## 3. Membrane Filtration Technology

Membrane filtration is an advanced drinking water treatment that is widely used nowadays in water treatment processes, mainly for drinking water. Examples of types of membranes include microfiltration (MF), ultrafiltration (UF), nanofiltration (NF), reverse osmosis (RO), electrodialysis (ED), forward osmosis (FO), and membrane distillation (MD). Each method has its own specific range of membrane pore sizes, surface charge, and hydrophobicity that is produced from different materials [39]. Table 1 shows the pore size ranges of various membrane filtration systems as compared to the size of common water contaminants.

**Figure 3:**
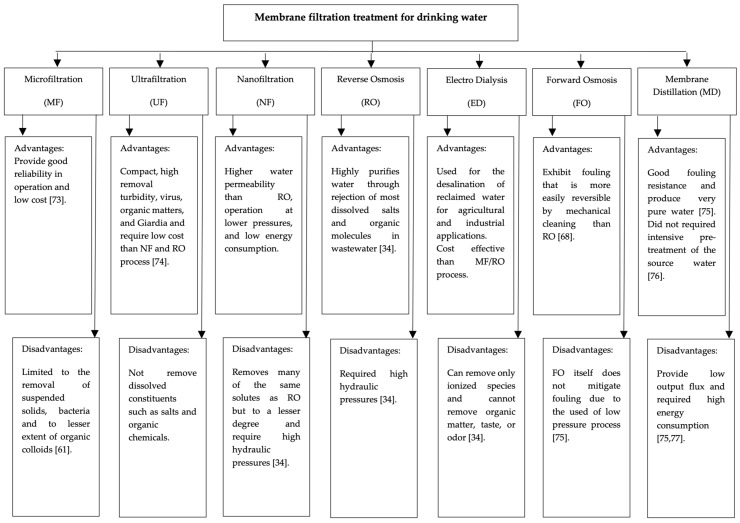
Available membrane filtration treatment for drinking water [34,61,68,72,73,74,75,76,77].

The application of membrane filtration technology to drinking water treatment on a large-scale [40] has received attention due to its advantages, including excellent effluent quality [41], simple process management [42], and strict solid-liquid separation with a small footprint requirement [43,44]. The technology is also easy to adapt to the existing treatment facilities [45], provides low energy consumption [11], and removes various contaminants [46]. The removal rate of contaminants depends on the characteristics of the membrane and the properties of the contaminant [36]. Aside from these benefits, the main disadvantage of this technology is the cost of the membrane itself, which can be reduced or eliminated if the membrane filtration process is handled properly. Figure 3 shows the advantages and disadvantages of each membrane filtration treatment applied to drinking water treatment.

In general, membrane filtration can be classified into two categories: low-pressure membrane (10 to 30 psi) and high-pressure membrane (75 to 250 psi). The low-pressure membrane system includes MF and UF, while NF and RO are categorized as high-pressure membrane systems.

The low-pressure MF and UF membranes for the application of municipal surface water treatment have been studied and implemented since the 1980s. In these studies, the MF (nominal pore size of 0.2 mm) and UF (nominal pore size of 0.01 mm) have proven their high capabilities for the removal of particulate matter (turbidity) and microorganisms [47,48]. MF and UF membranes were proven to provide a barrier to microorganisms such as Giardia cysts and Cryptosporidium oocysts, while the UF was proven to be an absolute barrier to viruses due to its smaller pore size of 0.01 mm [49,50]. Previous studies [51,52] also demonstrated that low-pressure membranes were able to treat turbidity efficiently using pilot and full-scale plants. The low-pressure MF and UF membrane systems provide high performance for the removal of contaminants from surface water, and other advantages include a smaller footprint, low chemical usage, and more automation. However, the limitation of membrane technology, including MF and UF, is the high cost of membrane replacement and the lower effectiveness in removing dissolved organic matter in the treated water. The study of modified MF membrane technology is reported by Sinclair et al. [53], and it showed an improvement in reducing cost as they do not require any external driving force. Unfortunately, the modification resulted in an approximately 22% loss of membrane permeability.

Meanwhile, He et al. [54] published a study on improved UF technology in which they combined heterogeneous catalytic ozonation and a UF membrane filtration technique for the long-term degradation of bisphenol A (BPA) and humid acid (HA). Results have shown improvements in removal efficiency, reduction of membrane resistance, and mitigation of membrane fouling. Another study concerning UF was reported by Chew et al. [55], which compared and evaluated industrial-scale UF with conventional drinking water treatment systems. The study showed that UF systems can provide reliable filtrate quality even with the existence of fluctuation in the raw water quality. In addition, the UF system offers promising sustainability, with no coagulant required for high-quality filtrate and non-toxic sludge discharge.

High-pressure NF and RO membranes can provide an alternative method for removing organic and inorganic matter. The NF process is already known for its capabilities in the removal of total organic carbon (TOC) in surface water treatment [56]. This process has been implemented in several drinking water industries [57,58,59]. In an experiment conducted using pre-ozonation as a pre-treatment process for NF membranes proposed by Vatankhah et al. [60], it was found that pre-ozonation with a low specific ozone dose could effectively mitigate a significant portion of fouling. However, the removal performance of dissolved organic carbon (DOC) of the NF membrane did not show a substantial change, which may be due to the relatively low applied ozone dose. The RO process is applied for drinking water treatment, whether the source water comes from seawater, brackish water, or groundwater [21]. However, RO has a problem with the ability of suspended solids, colloidal material, and dissolved ions in raw water to foul the system [61]. A study conducted by Touati et al. [62] combined UF, NF, and RO processes for isotonic and drinking water treatment. Results showed that the UF process used as pre-treatment was able to eliminate natural organic matter (NOM), while the NF process was able to characterize the fouling mechanism. The overall performance’s energy consumption is determined by salt rejection during the NF process.

Apart from RO, ED is another process that can be used to treat brackish water with high performance and energy efficiency [63,64]. The process involved the transfer of electrolytes or ions through a solution and membranes based on an applied electric field as the driving force [65]. Walha et al. [66] investigated the use of the NF, RO, and ED processes in producing drinking water from a brackish water source. The results showed the treatment based on RO and ED processes is more efficient, as shown by the high rejection of inorganic matters present in the feed waters. The concentration of ions in the permeate flux can achieve World Health Organization (WHO) standards, and it is more economical than the NF process.

Forward osmosis (FO) and membrane distillation (MD) processes are driven by heat, which is different from the pressure-driven process usually used for potable water reuse [67]. FO processing operates at low or no hydraulic pressure, which may reduce irreversible fouling and achieve high rejection of contaminants [68]. However, Li et al. [69] reported that the water flux produced by the FO process was still inadequate compared to the RO process under a similar applied pressure. FO processes involve a permeable membrane and two solutions, known as feed and draw solutions. The feed and draw solution consists of different concentrations that produce the osmotic pressure gradient that acts as the driving force for water permeation across a semi-permeable membrane [70]. An experiment conducted by Tow et al. [71] studied the fouling propensity between RO, FO, and MD. The experiment was conducted using a single membrane module and showed that both FO and MD exhibit a significant advantage in fouling resistance but neither of them performed well with both organic and inorganic foulants.

### Membrane Filtration and Fouling Issue

In membrane filtration, fouling is still the main reason for flux decline, and it needs to be reduced appropriately. Fouling is formed during the membrane filtration process. It is a very complex phenomenon that developed based on a combination of physical, chemical, and biological aspects. Membrane fouling can cause a reduction in permeate flux [78], an increment of trans-membrane pressure (TMP) [79], a shorter membrane life span [80], and consequently, cause a reduction of water quality [81]. Another work in [82] also claimed that membrane filtration is fraught with disadvantages regarding the amount of permeate flux and fouling tendency. Membrane filtration treatment is also struggling with a downside where it requires high operation and maintenance costs including labor, chemicals, membrane replacement, energy, and sludge disposal [83,84], when irreversible fouling on the membrane surface is not properly controlled.

Fouling takes place in the membrane filtration process based on four types of foulants: particulates/colloidal, organics, inorganics, and micro-biological organisms [85]. Table 2 illustrates each foulant type and its associated membrane fouling mode. Particulates or colloids with a similar or close diameter to the membrane pores can cause the membrane pores to become clogged, whereas larger particulates that are unable to pass through the membrane pores can cause the formation of a cake layer on the upstream face of a membrane. Organic and inorganic foulants tend to adsorb and precipitate in the membrane pores and consequently cause blockage of the membrane pores, whereas the accumulation of microorganisms on the membrane surface will cause the development of biofouling. Pore blocking, cake layering, adsorption and precipitation of organic-inorganic fouling, and biofouling occurrences cause a reduction in the rate of permeate production and escalate the complexity of the filtration process.

In general, there are two categories of fouling: reversible and irreversible fouling. The reversible fouling which is back washable and non-back washable occurs when organic or inorganic materials accumulate on either side of the membrane surface as operating time increases [86,87]. Back washable reversible fouling can be restored based on physical and hydrodynamic methods, while non-back washable reversible fouling can only be removed based on chemical cleaning. The irreversible fouling is usually occurring after quite a long run of filtration process where the particles formed a matrix that strongly attached to the membrane surface like pore blocking, clogging, biofilm, and cake gel [43,88]. Arise of irreversible fouling caused a loss in transmembrane flux. In this case, the membranes can only be fixed by extensive chemical cleaning. In the worst case, the membrane needs to be replaced.

The occurrence of membrane fouling in membrane filtration processes is due to many factors [89]. It is due to the characteristics of the feed water, membrane properties, and configuration of the filtration system itself [90]. Several studies on the factors that influence fouling have been conducted to control and mitigate its development. For example, Mozia et al. [91] showed the effect of process parameters which are feed cross-flow velocity and TMP, on the fouling behavior of the MF/UF system, whereas Kola et al. [92] discovered the fouling behavior for different feed water types and different membrane pore sizes. Results indicated that the parameters involved in both studies closely influence the fouling growth rate. In the work of Zhao et al. [93], the fouling was mitigated by controlling the membrane surface shear rate. The authors observed that by providing a high shear rate, the filtration process was able to achieve high critical flux. High shear rates cause algae to foul the membrane. This claim can be supported by similar research done by Jaffrin [94]. Table 3 tabularizes the parameters that influence membrane fouling during the filtration process. From the table, systematic approaches can be strategized to provide high-quality drinking water.

## 4. Current Solutions and Way Forward

Numerous fouling reduction techniques have been studied by many researchers to ensure the successful application of membrane filtration systems. In this review, the fouling reduction methods proposed by the previous researchers can be classified into three main categories: chemical cleaning, physical cleaning, and hydrodynamic cleaning [111], as summarized in Table 4. Chemical cleaning is a process that is usually used as a pre-treatment method. The process is recognized as a prevention method. It involved chemical agents as a tool to reduce or eliminate the deposition of fouling. Reversible fouling based on the natural organic matter can be partially or fully restored by chemical cleaning. Reversible fouling can also be removed physically. Irreversible fouling can only be removed by chemical cleaning. In general, chemical cleaning is executed when physical cleaning no longer provides effective cleaning performance and the flux cannot restore the environment sufficiently. However, the cleaning method for each filtration process is dependent on many factors. Still, trial-and-error practice is the most suitable method to get the best strategy for any process.

**Figure 4:**
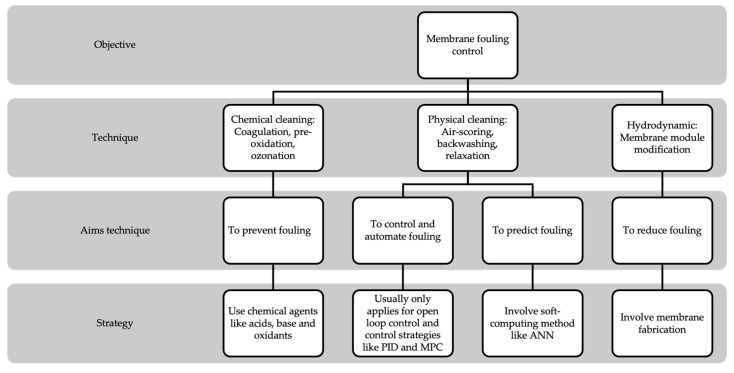
Membrane fouling control.

In this review, different views and perspectives on fouling reduction methods are discussed as a way forward to solving the issue, which are prevention, prediction, or control automation. Figure 4 summarizes the main strategies that were used for membrane fouling control. The prevention method is usually related to chemical cleaning, while the prediction and control automation methods are related to physical cleaning. The hydrodynamic technique involves modification of module design and arrangement of flow such as for feed and permeate. The hydrodynamic technique has been studied by Lee et al. [112] to control the fouling during the forward osmosis-reverse osmosis (FO-RO) hybrid process. The study evaluated the influence of feed flow rate, draw flow rate, and hydraulic pressure difference. The results showed that the high feed flow rate was able to effectively mitigate the fouling. The high draw flow rate, on the other hand, causes an increase in the fouling growth rate. In addition, increasing hydraulic pressure does not affect reducing the fouling growth rate.

**Table 4:** Fouling reduction techniques.

Reference	Filtration Type	Plant Size	Fouling Reduction Technique
H. Rho et al. [113]	UF	Pilot	Chemical
R. Bert et al. [114]	UF	Lab	Hydrodynamic
S. Kim and C. Park [115]	UF	Bench	Chemical
C. Lee et al. [112]	FO-RO	Pilot	Hydrodynamic
K. Almoalimi and Y. Liu [116]	FO	Lab	Physical
B. Unal [117]	RO	Full-scale	Chemical
L. Martinelli et al. [118]	UF	Lab	Hydrodynamic
M. Yang et al. [119]	MBR	Lab	Hydrodynamic
H. Jang et al. [108]	UF	Lab	Chemical
I. Ruigómez et al. [120]	UF	Lab	Physical
W. Zhang et al. [121]	AGS-MBR	Lab	Hydrodynamic
W. Yang et al. [122]	UF	Pilot	Chemical

There are also efforts to improve methods by optimizing operational conditions [123,124], both in chemical and physical cleaning operations. To optimize operating conditions, it is important to understand the characteristics of accumulated irreversible fouling. The irreversible fouling is a very complicated phenomenon in which membrane characteristics (membrane materials, pore size, configuration, hydrophobicity, charge), process operating conditions (TMP, temperature, permeate flux), and influent physicochemical properties (particle size distribution, inorganic or organic mattes) closely influence each other. Soft computing optimization is one of the best solutions to handle complex and nonlinear processes.

### 4.1. Fouling Prevention

Fouling prevention is important in order to prevent fouling (reversible or irreversible) from occurring or arising. In the prevention step, the foulants that can cause fouling are eliminated before the feed water enters and passes through the membrane [125]. In practice, an irreversible type of fouling is removed using chemical cleaning methods [126]. For safer operation, acids, bases, and oxidants are usually used in chemical cleaning [127,128]. The quantity of chemical cleaning is monitored to avoid excessive chemical use that can damage the membrane surface and increase the cost of operations [129]. Therefore, it is important to optimize the operating conditions of chemical cleaning, which involve cleaning intervals, cleaning duration time, chemical type, and chemical concentration. Yoo et al. [3] showed that proper optimization of operating conditions for chemical cleaning was able to reduce energy consumption, chemical use, and sludge production. However, the process causes an increase in the membrane replacement cycle.

Previous researchers applied numerous techniques as a pre-treatment method of preventing fouling from occurring, such as coagulation [130], oxidation, ozonation, and adsorption methods [73]. The coagulation process disperses and suspends contaminants and is suitable for natural organic matter (NOM) with a high molecular weight [131,132]. The process commonly uses pre-hydrolyzed salts such as polyaluminium and sulfates as coagulant agents. The process required low cost due to the simple operation, conversely it produces high sludge formation. Unlike coagulation, the oxidation process produces a lesser amount of sludge formation. The process is useful for the removal of dissolved organic contaminants such as arsenic and humic acid [133], the same as the ozonation process [60,134]. The main advantage of ozonation treatment is that it does not produce any sludge, but it can cause the degradation of biopolymers and high energy consumption. The adsorption process is frequently used to remove organic and inorganic micro-contaminants from pharmaceuticals and personal care products, such as pesticides, antibiotics, detergents, soaps, and oils [135]. The process is drive by the electrostatic interaction of negative and positive charged that produce between influent and adsorbent [136]. The influent, which is a liquid/water or gaseous contaminant, will change into solid formation [137,138]. The absorbent can be restored and reused. The process is capable of removing micro-contaminants even when they are present in trace amounts in water. Adsorption process is more efficient when ozonation is used as a pre-treatment.

Oloibiri et al. [139] show that discovered that combining pre-treatment methods yields better results in reducing fouling tendency than a single pre-treatment method [140]. Yu et al. [125] studied the effect of the conventional coagulation technique and hydrogen peroxide (H_2_O_2_) addition at three doses level during the backwash process. The performance of the technique was measured based on the rate of TMP. The authors found that the addition of H_2_O_2_ at all doses was able to prevent any measurable increase in TMP, which represents the success of the proposed technique to prevent the development of membrane fouling. Wang et al. [141] used H_2_O_2_ in the pre-oxidation process before executing the coagulation process. In this study, the TMP value, microorganism development, and cake layer rate were monitored to observe the effect of biofouling in the presence of H_2_O_2_. Results showed that the proposed technique was able to decelerate the microorganisms’ growth rate and reduce the cake layer, hence decreasing the TMP value, which indicated a reduced membrane fouling tendency.

Park et al. [142], investigated a pre-ozonation technique based on two doses for the NF process’s surface water brine. The technique was mainly applied to reduce or control membrane fouling, where the doses are determined based on the residual ozone dose. A precise ozone dose is required to avoid membrane damage and an increase in operating costs. The authors observe that the pre-ozonation technique was able to reduce a significant amount of organic fouling potential with relatively low ozone doses. Results also indicated that the applied technique was able to act as a barrier for the removal of trace organic compounds which are important for water treatment.

Another study on the pre-ozonation technique as a membrane fouling prevention method was reported by Wang et al. [143]. In this work, the effects of pre-ozonation as a pre-treatment for the UF process on secondary wastewater effluents are investigated. The research is based on two types of UF membranes: hydrophilic regenerated cellulose membranes and hydrophobic polyethersulfone membranes. The result showed that high fouling reduction was attained for the hydrophobic membrane at high ozone doses. Table 5 presents the settings for membrane fouling prevention from previous researchers. The results from the previous studies cannot be generalized because the result and consequence of each pre-treatment method are expected to diverge according to the feed water, filtration technology, and pre-treatment material, such as the types of absorbent and oxidation agent.

### 4.2. Fouling Prediction

Many researchers are interested in foul prediction. The prediction will be able to help the researchers forecast the best operating conditions for a particular process and determine the parameter that can trigger the fouling. It is also useful to circumvent or slow down the process of fouling developments. The prediction process is a part of modeling and controlling development. Some researchers used prediction terms to describe the modeling process, which can be categorized as mathematical and empirical processes.

As mentioned previously, the prediction or modeling of physical cleaning operations has been widely utilized by researchers to understand fouling behavior. Physical cleaning operations include air scouring, backwashing, and relaxation operations. Air scouring, also known as aeration or air bubble control, is a widely used method of membrane cleaning. The method boosts the saturation of oxygen by applying air bubbles that exhibit cross-flow velocity and can eliminate reversible fouling [150]. The backwashing process involves the pumping of permeate or water backward through the filtration module (membrane) in order to remove the particles attached to the membrane surface. Other than permeate, the backwash can be implemented using either chemicals, clean water, or air. Finally, relaxation is a process where the permeating or filtration process is temporarily idle, but with the air bubbles scouring continuously working to relieve the membrane from the generated pressure [151,152]. A comparative study of physical cleaning involving air scouring, backwashing, and relaxation techniques to control the fouling in drinking water treatment was conducted by De Souza and Basu [153]. In this study, it was shown that in some cases, backwashing and relaxation durations have integrated results for the reduction of fouling, while air scouring can reduce fouling at the highest level with the highest air scour rate. Overall, the result indicated that the combination of the three techniques outperformed air-assisted backwashing alone in terms of fouling reduction. It is crucial to understand the effectiveness of each operation (air scouring, backwashing, and relaxation) when controlling membrane fouling in order to properly strategize the coordination of the operations. Fouling may also be controlled by operating UF under its critical flux [26]. When UF is operated under its critical flux, foulant deposition on the membrane surface can be avoided. Thus, membranes can be operated with a stable flux. Vigneswaran et al. [154] also mentioned that the performance of the membrane combined with the adsorption process is influenced by the reactor configuration, mode of operation, carbon dosage, adsorption, and influent characteristics.

Work by Kovacs et al. [155] proposes a mathematical framework for batch and semi-batch modeling techniques for membrane filtration processes. The proposed method uses feed concentrations as the basis for calculation and can be applied to all pressure-driven membrane filtration processes. The main advantage of the proposed method is that it can capture the dynamic behavior of all types of batches and semi-batch configurations without changing the general mathematical framework. However, it required challenging mathematical problem-solving to obtain the general framework, whereas Ghandehari et al. [156] proposed a semi-empirical and artificial neural network (ANN) modeling technique to predict the characteristics of microfiltration systems based on permeate flux decline and membrane rejection. Results showed that the semi-empirical method was able to predict the flux only for a specific time, unlike the ANN method. The ANN method can model the membrane filtration system over the entire filtration time for all tested operating conditions.

Ling et al. [157] proposed a tent sparrow search algorithm back propagation network (Tent-SSA-BP) technique for predicting membrane flux in a membrane bioreactor (MBR) fouling model. They utilized the principal component analysis (PCA) algorithm to reduce the initial auxiliary variables. A study was conducted to compare the genetic algorithm back propagation (GA-BP), particle swarm optimization back propagation (PSO-BP), sparrow search algorithm extreme learning machine (SSA-ELM), sparrow search algorithm back propagation (SSA-BP), and tent particle swarm optimization back propagation (Tent-PSO-BP) networks. The results indicated that the Tent-SSA-BP technique provided the best performance in terms of training speed and prediction accuracy. The Tent-SSA-BP technique predicts with 97.4% accuracy, whereas BP predicts with only 48.52% accuracy. A model for MBR prediction has also been studied by Kovacs et al. [158], where it predicts transmembrane pressure (TMP) at various stages of the MBR production cycle. The prediction was performed based on a data-driven machine learning technique involving a random forest (RF), artificial neural network (ANN), and long-short-term memory (LSTM) network. Among the proposed methods, RF models provide the best statistical measures. The obtained prediction models produce promising results, but their ability to predict the data is limited at this time. Yao et al. [159] predict the variation of the TMP in the constant flux mode by proposing a novel method based on the loss of effective filtration area. The result showed a high correlation coefficient, which indicates a good model prediction.

Another study on fouling prediction was conducted by Chew et al. [160], where the first principle equation of Darcy’s law on cake filtration and ANN were combined to predict the models that represent the dead-end ultrafiltration process. In this study, the turbidity of the feed water, filtration time, and TMP were used as the input parameters. The sensitivity analysis showed that there was a strong linear correlation between specific cake resistance and turbidity. The proposed models can predict the specific cake resistance and total suspended solids (TSS) of feed water with high accuracy, which provide an early indication of fouling development.

Another study on fouling prediction was reported by Lie et al. [161]. In this work, experiments were conducted on a constant flow microfiltration membrane system at critical flux and supra-critical flux conditions with various permeate fluxes and feed water qualities. In this study, five input variables of the ANN model, including permeate flux, turbidity, UV_254_, time, and backwash frequency were used for the prediction of TMP. The results show that the ANN model with five input parameters can predict TMP behaviors, where the TMP value is used to indicate fouling propensity. A similar study using ANN models was done by Hazrati et al. [162], where back propagation algorithms were used to predict the effluent chemical oxygen demand (COD) and TMP. The research indicated that the ANN model can easily be used to predict the concentration of COD and TMP in effluent. The study also investigated the specifications of the cake layer at different hydraulic retention times (HRTs) in order to control membrane fouling. Results indicated a linear relationship between the reduction in HRT and the particle size of the cake layer.

An ANN technique was also found by Abbas and Al-Bastaki [163], where an experiment was conducted using a spiral wound reverse osmosis membrane system with three operating conditions of inputs were studied. The first one was trained using a total of sixty-three data points from different operating temperatures for training purposes. The second one is trained only using forty-two data points corresponding to the operating temperatures of 10 °C and 30 °C; another twenty-one data points corresponding to the operating temperature of 20 °C were employed for testing purposes. The third condition was trained using the data corresponding to the operating temperatures of 10 °C and 20 °C, whereas another 21 data points corresponding to the operating temperature of 30 °C were employed for testing purposes. It was found that ANN was able to interpolate the data with good accuracy but was unable to produce acceptable results for data extrapolation, whereby works by Chen and Kim [164] used 17% of experimental data for training purposes and 83% for verification. The authors studied the capability of a radial basis function neural network (RBFNN) and a multilayer feed-forward backpropagation neural network (BPNN) to predict the permeate flux in cross-flow membrane filtration. The predictions are based on five input parameters, which are particle size, ionic strength, pH, TMP, and elapsed time. The result shows that a single RBFNN is able to predict the permeate flux and provide better predictability than a BPNN.

Based on the review conducted, it was found that fouling prediction has been broadly applied for the mitigation of membrane fouling. Various techniques have been implemented, but most of them apply ANN as a primary strategy. Due to the difficulty of solving tricky mathematical problems, only a few studies use mathematical frameworks in prediction. Many of them also combine the ANN technique with other methods. The ability of ANN to solve highly complex and nonlinear problems makes it extremely useful in the treatment of drinking water. ANN is capable of providing good predictions even without detailed information about the physical parameters of the system, relying solely on input-output data. Even so, the process of determining appropriate input-output parameters is crucial and plays a significant role. Without a good relationship between the selected input and output parameters, acceptable prediction cannot be achieved. Therefore, it is important to decide the respective input-output parameter before proceeding with the ANN architecture. Every process comes with differences and complex characteristics that are likely due to the system itself. Certainly, changing the concentration of the feed water will change the entire process. As a result, understanding the process in terms of which parameter caused an effect on which parameter is critical in the ANN technique. Table 6 shows the various ANN settings for the prediction of membrane fouling in drinking water treatment. Based on Table 6, it is clearly shown that each system with different feed water characteristics involves different input and output parameters. Hence, this will affect the ANN architecture. 

### 4.3. Fouling Control and Automation

To overwhelm the problem that fouling causes, it is essential to equip the membrane filtration process with an effective controller. The effective design of the controller will be able to improve the overall efficiency, increase the membrane’s lifespan, and reduce the total operating costs. However, the design of the controller is not an easy task due to the many impediments, such as the dynamic processes of the system itself, the difficulty of modeling the system, variations in feed water quality, system faults, membrane fouling, and the requirement of continuous monitoring for membrane cleaning.

As the system becomes more complex, the control strategies make it easier to handle the membrane filtration process by estimating the uncertainties and making control systems that are robust and reliable. However, based on the literature, there is still a lack of research that applies control automation to the membrane fouling problem [171]. Most of the previous research focused on open-loop control, membrane modifications, physical cleaning, and pre-treatment methods.

In the previous study on control automation, Azman et al. [172] applied a proportional-integral-derivative (PID) controller with the Ziegler Nichols (ZN) and Cohen-Coon (CC) tuning methods for the coagulation and flocculation filtration processes. The robustness of the controller’s performance was measured based on the step test, set point change, and load disturbance test. At the end of the study, it was shown that the PID controller with the ZN tuning method exhibits better performance than the PID controller with the CC.

The design of model predictive control (MPC) based on a support vector machine (SVM) model for the ozone dosing process is reported by Dongsheng et al. [173]. The results have shown an improvement in maintaining a constant ozone exposure compared to the use of the proportional-integral (PI) controller. However, the controller design was only tested for a plant-scale experiment. The design of MPC was also found in the works by Bartman et al. [174], where the purpose was to determine and control the optimal switching path of flow operating conditions, thereby reducing the fouling problem for a RO desalination process. Results showed that the proposed controller was able to reduce the variation of system pressure, and hence, provide smaller pressure fluctuations with a shorter transition time. The designed MPC can control and prevail over the disturbance that comes through the system and reduce the percentage error between the actual and the desired final steady-state value.

Multiple model predictive control (MMPC) was used in the simulation works of Bello et al. [175] to control and optimize the amount of chemicals used in the coagulation process of water treatment plants. They applied switching mechanisms to deal with the control input constraints explicitly. Simulation results show that the proposed MMPC provides better performance than conventional control. However, the work is only conducted based on the linear model; future work may use the nonlinear model, which represents the real system. Rivas-Perez et al. [176] designed an expert model of predictive control (EMPC) to control the critical variables of the pilot scale RO desalination plant. Based on known information, an expert system was created that can lead to decision-making strategies. The robustness of the proposed controller was evaluated based on two real-time cases. In the first case, the performance of EMPC and the ability to ignore disturbances were tested. In the second case, the performance of the proposed EMPC was compared to the performance of the standard MPC. The results showed that the control plant with EMPC provided higher accuracy and robustness than standard MPC, especially for time-varying parameter rejection. Table 7 shows the modeling and control strategy that has been reported based on several techniques by the previous researcher to maximize and control the quality of drinking water treatment.

## 5. Conclusions

This paper summarizes the available filtration treatments for treating drinking water. Filtration treatment can be categorized into two main types, i.e., conventional and advanced treatment. As discussed in the relevant section, conventional treatment entailed additional costs due to the need for additional treatment and a large footprint, whereas advanced treatment, specifically membrane filtration treatment, is now well-established in the industry because it is capable of overcoming the disadvantages caused by conventional treatment. Membrane filtration treatment achieves satisfactory results in the elimination of different kinds of contaminants from effluent. As a result, the rate of permeate flux (effluent) production will increase. However, membrane filtration is facing problems with membrane fouling as the operating time increases.

Until now, many researchers’ studies on the parameter that causes fouling have resulted in the development of a model for prediction and prevention. Membrane fouling is affected by many factors, including feed water type, feed water and membrane properties, membrane material, filtration strategy, and process operating conditions such as transmembrane pressure and sludge retention time. Previous studies showed that membrane fouling in processes can be very diverse, and it is mainly due to the feed water type and the process treatment itself. In this case, understanding the composition of the feed water and the characteristics of the process treatment are crucial. Fouling mitigation is typically based on prevention, prediction, and control automation process. The prevention method has been utilized broadly and presents promising results for water treatment. The procedure involving the use of chemicals as an agent to mitigate fouling is the method’s main shortcoming. Since the process discussed drinking water, which is closely related to human health, it is remarkable to prevent any approach that could cause undesirable consequences. For the prediction method, a former researcher mostly applied ANN as a tool to predict the development of fouling. Conversely, the study did not discuss in detail the technique to reduce fouling but instead focused only on prediction purposes. Nevertheless, there is not much information presented on control automation strategies. The majority of researchers control membrane fouling through pre-treatment or modification of membrane characteristics, both of which required the use of chemicals. It is critical for ecologically mitigating membrane fouling. Future research is needed to add value to the control automation method via the application of control strategies such as controllers (proportional-integral-derivative controllers, model predictive controllers, etc.). A study on membrane automation is necessary to control the occurrence of fouling without the use of chemical agents. It is thought that this will lead to more exciting discoveries, directly encounter fouling, and produce high-quality drinking water. In the years ahead, it might be switched to fresh strategies and technologies.

## Figures and Tables

**Table 1:** Contaminant with respective membrane filtration type.

Size (mm)		0.0001	0.001	0.01	0.1	1.0	10	100	1000
	Filtration Type	Reverse osmosis							
		Nanofiltration							
			Ultrafiltration						
Contaminant					Microfiltration				
							Conventional		
Metal Ions		√	√						
Aqueous Salts		√	√						
Humic Acids			√	√					
Viruses				√					
Clays					√	√			
Assestor Fiber					√	√	√		
Bacteria					√	√	√		
Cycst						√	√		
Algae						√	√	√	
Sand								√	√

**Table 2:** Types of foulant.

	Types of Foulant			
	Particulates/Colloidal	Organics	Inorganics	Micro-Biological Organisms
Example	Organic and inorganic particles like corrosion products, sand, and clay.	Dissolved components of natural organic matter (NOM) like proteins, carbohydrates, and humid acid.	Dissolved components like iron, silica, metal oxides, calcium phosphate, and aluminium hydroxides.	Viruses, bacteria, algae, fungi, and microorganisms.
Modes of membrane fouling	Development of pore blocking and formation of cake layer that can physically blind the membrane surface.	Foulant will adsorb to the membrane.	Foulant will precipitate on the membrane surface.	Arise of biofouling either by attachment and/or growth.

**Table 3:** Parameter that influences the fouling growth rate.

Reference	Parameter Influence Fouling	Example
J. Li et al. [95], W. Zhang and T. Hao [96]	Feed water type	River, lake, sea, raw, synthetic, micro-polluted, municipal WWTP
H. Zhang et al. [97], L. Wang et al. [98], Z. Pan et al. [99], Q. Gao et al. [100]	Feed water properties	Particle size distribution, colloidal, organic, and inorganic matter
Y. Li et al. [101], F. Zhao et al. [102], H. Lay et al. [103]	Membrane properties	Pore size distribution, pore shape, surface and bulk porosity, thickness, surface charge, contact angle, surface roughness, hydrophobicity, shear rate
C. Charcosset [104], H.He et al. [105]	Membrane material	PVDF, PTFE, acrylic copolymer, nitro-cellulose, cellulose acetate, nylon, polycarbonate
H. Liu et al. [106], M. Enfrin et al. [107], H. Jang et al. [108]	Filtration strategy	Submerged, crossflow, chemical cleaning (acids, bases, oxidants), physical cleaning (air scouring, backwashing, relaxation), hydrodynamic method, optimize the operating condition
N. Park et al. [109], L. Nthunya et al. [110]	Process operating condition	TMP, temperature, permeate flux, feed cross-flow velocity, sludge retention time, hydraulic retention time, turbidity

**Table 5:** Setting of membrane fouling prevention in drinking water treatment.

Reference	Feed Water	Filtration and Membrane Type	Measured Parameter	Applied Technique
Yu et al. [125]	Synthetic raw water	UF-Hollow fiber	TMP, EPS (protein and polysaccharide), the fluorescence of organic matter	Coagulation pre-treatment and addition of hydrogen peroxide (H_2_O_2_) during the backwash process
Wang et al. [141]	Mix of domestic sewage and tap water	UF-Hollow fiber	TMP, microorganisms, EPS (proteins and polysaccharides)	Pre-oxidation of H_2_O_2_ and coagulation of aluminum sulfate
Ma et al. [144]	Raw water from the reservoir	UF-Hollow fiber	TMP, flux, turbidity, chromaticity, the concentration of Mn and Fe	Pre-oxidation of KMnO_4_-Fe(II) and compared to coagulation of Fe(III)
Xing et al. [145]	Raw water from the reservoir	UF-Hollow fiber	TMP, irreversible TMP, DOC, ammonia (NH4^+^_-_N), UV_254_, turbidity, the fluorescence of organic matter, disinfectant curve	Combination of polyaluminium chloride (PACl) coagulation-sedimentation and powdered activated carbon (PAC) adsorption
Guo et al. [146]	Raw water from the river	UF-Hollow fiber	TMP, DOC, NH4^+^_-_N, UV_254_, COD_Mn_	Coupled continuous sand filtration (CSF) and UF process
Imbrogno et al. [147]	Natural/ pure water (pH8)	NF-Flat sheet	Flux, irreversible flux	Combination of magnetic ion exchange resins (MIEX) and NF in one single process
Tian et al. [148]	Raw water from the river	UF-Hollow fiber	TMP, DOC, fluorescent spectrum, molecular weight distribution, hydrophobicity	Pre-oxidation of ultraviolet/persulfate (UV/PS)
Cheng et al. [149]	Raw water from the river	UF-Not mentioned	Flux, fouling resistance	Pre-ozonation with three different doses

**Table 6:** ANN setting for the prediction of membrane fouling in drinking water treatment.

Reference	Feed Water	Filtration and Membrane Type	Input Parameter	Output Parameter	Model/Training	Activation/Layer/Performance
Liu et al. [161]	Three types of synthetic water	MF-Hollow fiber	Permeate flux, feed water turbidity, UV_254_, operating time, backwash types	TMP	LM/BP	Sigmoidal/ 4/ R^2^ = 0.98
Schmitt et al. [165]	Domestic wastewater	RO-Flat sheet	pH, alkalinity, MLSS, COD, total nitrogen, ammoniacal nitrogen, nitrate, total phosphorus, DO, MLVSS	TMP	LM/BP	Sigmoidal/ 3/ R^2^ = 0.850
Mirbagheri et al. [166]	Wastewater treatment plants	UF-Hollow fiber	Operational time, TSS, influent COD, SRT, MLSS	TMP and membrane permeability	MLP/RBF/BP/BBP/LM	Radially symmetric basis/ 3/ Perfect match
Shetty and Chellam [167]	Ground and surface waters of eleven sources	NF-Flat sheet and spiral wound	Operational time, influent water flow rate, pH, feed water TDS concentration, UV_254_, permeate water flux or TMP, temperature, feed water flow rate	Membrane resistance	MLP/ LM	Sigmoidal/ 3/ RE = 5%
Delgrange et al. [168]	Natural water	UF-Hollow fiber	Permeate flow rate, turbidity, turbidity previous cycle, mean TMP at filtration start, mean TMP before previous backwash	TMP	QN	Sigmoidal/ 3/ Good accuracy
Zhang et al. [6]	Monthly data from 45 DWTPs across China	–	Temperature, COD of raw water, total electricity consumption, turbidity, NH_4_, pH, residual free chlorine of treated water, lime hydrate dosage, PAC, active chlorine, the tertiary process cost	Monthly average drinking water production (m^3^)	ENN/FNN/LM/BR/QN/GD/OSS/RP	Genetic algorithm/ 3-5/ R^2^ = 0.93
Li and Wang [169]	Wastewater	–	MLSS, operating pressure, total resistance, pH, COD, temperature	Membrane flux	ENN/BP/GD	Sigmoidal/ 3/ RE = 5.8%
Cai and Li [170]	Sewage treatment plant	–	MLSS, total resistance, operating pressure	Membrane flux	WNN/BP/GC	Morlet/ 3/ RE = 3.8%

**Table 7:** Setting of control strategy in drinking water treatment.

Reference	Filtration Type	Modeling/Control Strategy	Control Parameter	Manipulate Parameter
Bello et al. [175]	Coagulation	Differential and algebraic equations/ Multiple MPC	Surface charge and pH	Chemical reagents flow rates
Rivas-perez et al. [176]	RO	Systems identification tools/Expert MPC	Permeate flow rate and permeate conductivity	Feed pressure and brine flow rate
Chew et al. [177]	UF	ANN predictive model/ANN	Filtration and backwash time	Turbidity, specific cake resistance, TMP, reverse TMP, and backwash water volume
Dongsheng et al. [173]	Ozonation	Support vector machine model/ MPC	Ozone dosing	Dissolved ozone residual
Bartman et al. [174]	RO	Overall mass balance and local energy balances/MPC	Pressure	Retentate and bypass stream velocities
Gil et al. [76]	Solar membrane distillation	Lumped-parameters Model/ MPC	Temperature and flow rate	Frequency
Azman et al. [172]	Coagulation and flocculation	First order plus dead time/PID	Turbidity	Voltage

## Data Availability

Data sharing not applicable.
